# REPLY TO THE AUTHORS: Re: Ureteroinguinal hernia with obstructive urolithiasis

**DOI:** 10.1590/S1677-5538.IBJU.2019.0415.3

**Published:** 2021-02-03

**Authors:** JuliAnne R. Rathbun, Nanda Thimmappa, Stephen H Weinstein, Katie S. Murray

**Affiliations:** 1 University of Missouri School of Medicine Division of Urology Columbia Missouri USA Division of Urology, University of Missouri School of Medicine, Columbia, Missouri, USA;; 2 University of Missouri School of Medicine Department of Surgery Columbia Missouri USA Department of Surgery University of Missouri School of Medicine, Columbia, Missouri, USA;; 3 University of Missouri School of Medicine Department of Radiology Columbia Missouri USA Department of Radiology, University of Missouri School of Medicine, Columbia, Missouri, USA

*To the editor*,

Although there are no known risk factors for uretero-inguinal hernias we note that BMI could attribute and this patient had a BMI of 45, classified as morbid obesity (1, 2). There are other cases of this rare hernia that were also observed in males with extremely high BMI (3). Management of any obstruction can be a challenge in this individual with anatomic complexity and other options may need to be considered including longer stent lengths and even antegrade ureteral access via nephrostomy tube. This patient was successfully managed with retrograde ureteroscopy with holmium laser lithotripsy of his stone and placement of a 6 French x 30 cm double-J ureteral stent. This was the longest stent size available and it was unable to completely reach the renal pelvis but was left for 3 days post-operatively to prevent distal ureteral obstruction after procedure. Although it is a rare occurrence, the availability of extra-long stents in hospitals may be necessary. To date, there has been no recurrence of urolithiasis for the patient but continued monitoring with intermittent imaging will be a necessity.

The Authors

## References

[1] 1. Khattak A, Feyisetan O, Floyd MS Jr, Samsudin A. Re: Uretero-inguinal hernia with obstructive urolithiasis. Int Braz J Urol. 2021;47:222-3. 10.1590/S1677-5538.IBJU.2020.0570 PMC7712706 33047938

[2] 2. Rathbun JR, Thimmappa N, Weinstein SH, Murray KS. Ureteroinguinal hernia with obstructive urolithiasis. Int Braz J Urol. 2020;46:857-8. 10.1590/S1677-5538.IBJU.2019.0415 PMC7822378 32648431

[3] 3. Won AC, Testa G. Chronic obstructive uropathy due to uretero-inguinal hernia: A case report. Int J Surg Case Rep. 2012;3:379-81. 10.1016/j.ijscr.2012.04.004 PMC3376670 22609811

